# Fully Controllable Pancharatnam-Berry Metasurface Array with High Conversion Efficiency and Broad Bandwidth

**DOI:** 10.1038/srep34819

**Published:** 2016-10-05

**Authors:** Chuanbao Liu, Yang Bai, Qian Zhao, Yihao Yang, Hongsheng Chen, Ji Zhou, Lijie Qiao

**Affiliations:** 1 Key Laboratory of Environmental Fracture (Ministry of Education), University of Science and Technology Beijing, Beijing 100083, China; 2 State Kay Laboratory of Tribology, Department of Mechanical Engineering, Tsinghua University, Beijing 100084, China; 3 State Key Laboratory for Modern Optical Instrumentation, Zhejiang University, Hangzhou 310027, China; 4 State Key Laboratory of New Ceramics and Fine Processing, Tsinghua University, Beijing 100084, China

## Abstract

Metasurfaces have powerful abilities to manipulate the properties of electromagnetic waves flexibly, especially the modulation of polarization state for both linearly polarized (LP) and circularly polarized (CP) waves. However, the transmission efficiency of cross-polarization conversion by a single-layer metasurface has a low theoretical upper limit of 25% and the bandwidth is usually narrow, which cannot be resolved by their simple additions. Here, we efficiently manipulate polarization coupling in multilayer metasurface to promote the transmission of cross-polarization by Fabry-Perot resonance, so that a high conversion coefficient of 80–90% of CP wave is achieved within a broad bandwidth in the metasurface with C-shaped scatters by theoretical calculation, numerical simulation and experiments. Further, fully controlling Pancharatnam-Berry phase enables to realize polarized beam splitter, which is demonstrated to produce abnormal transmission with high conversion efficiency and broad bandwidth.

Metasurface consisting of assembling arrays of sub-wavelength scatters has attract much attention in recently years as providing abrupt phase shift, amplitude modulation and polarization change of electromagnetic wave (EMW). By properly arranging the scatter distribution which can be treated as secondary point sources at surface, we can reshape the wavefront and realize many functionalities in ultrathin devices, such as broadband quarter-wave plate^1^,^2^, aberration-free lens^3^,^4^, invisibility cloak^5^,^6^ and optical holograms^7^,^8^,^9^. Besides, metasurface can also be integrated with different type of sensitive materials^10^,^11^,^12^,^13^,^14^ and demonstrate a more broad application prospect in constructing tunable devices, such as ultrafast sensor^11^,^13^, electro-optical switch^15^ or programmable logic component^16^.

For exotic applications of metasurface, it is important to modulate the polarization state of EMW for full 2π phase control, including conversion and rotation, and some examples have been successfully made to polarized beam splitter^17^,^18^,^19^, vortex beam^17^,^19^,^20^ and Bessel beam generator^21^,^22^. Especially, some metasurfaces with uniaxial birefringent scatters serving as half wave plates are designed to convert the handedness of circularly polarized (CP) wave to the opposite (cross-polarization) based on Pancharatnam-Berry (P-B) phase^23^. Compared with linearly polarized (LP) wave with tendency of polarization deflection and energy loss, CP wave has much better capability of anti-jamming due to the polarization isolation between incident wave and reflected wave, and its modulation by metasurface shows more broadband dispersionless properties and multi-functionality^19^,^24^,^25^, as the phase shift of cross-polarization depends on scatter’s rotation and handedness of CP wave while independent of incident wavelength.

As the striking features mentioned above, intensive researches are carried out to develop metasurfaces modulating CP wave. Ding *et al*.^24^ reported an ultrathin, Babinet-inverted metasurface for converting the polarization of CP wave and proposed planar metalens. Huang *et al*.^25^ realized helicity-dependent surface plasmon polariton unidirectional excitation by an array of elongated apertures. Pu *et al*.^26^ demonstrated semi-continuous catenary metasurfaces which could be used as vortex beam and high order Bessel beam generators. Generally, high polarization conversion efficiency, broad bandwidth and pure CP wave output are strongly desired for better performance of transmission type metasurfaces, but the previous results are far from satisfying. No matter for LP wave or CP wave, the transmission efficiency of cross-polarization conversion by single-layer non-magnetic or metallic metasurface have a low theoretical upper limit of 25% 24,27,28 and the experiment results are always even lower as a few percent^17^,^18^,^19^,^25^, whose low value severely works against the application requirements. In addition, the bandwidth is usually narrow and the purity of CP wave output is low, i.e. there is a large amount of co-polarization (same handedness as incident wave, 0-π phase coverage) remnant after transmission^19^,^24^,^25^,^26^,^28^.

Since single-layer metallic metasurface cannot completely control phase while maintaining a high conversion efficiency, some researchers adopted all-dielectric metasurface^22^ or multilayer structure^29^,^30^ to overcome the shortcomings due to an additional magnetic response from Mie scattering or loop current by cascading metallic layers, leading to near unit transmission and full phase coverage. These reflectionless metasurfaces can be designed by the Huygens’ principle^29^,^30^,^31^ or rigorous filter theory^32^. However, there are still problems for both methods. For all-dielectric metasurface, the requirement of matching between localized Mie-type electric and magnetic responses limits the bandwidth with high transmission, while multilayer structures always have too complicated metallic patterns or too many layers with different shapes^30^,^33^. Therefore, it is still challenging to construct a low profile metasurface which can fully control polarization and phase shift with high conversion efficiency in a broad bandwidth.

In this letter, we design a rather simple multilayer metasurface array relying on the interference of polarization couplings in the multi-transmission process resulting in enhancement for the transmitted fields of CP cross-polarization and reduction for other scattering fields. A high conversion coefficient is realized in a broad bandwidth with fully controllable phase, and the purity of CP wave output is high. Basically, a gradient metasurface array is demonstrated to bend the transmitted beam in arbitrarily trajectory.

## Results

**Figure 1:**
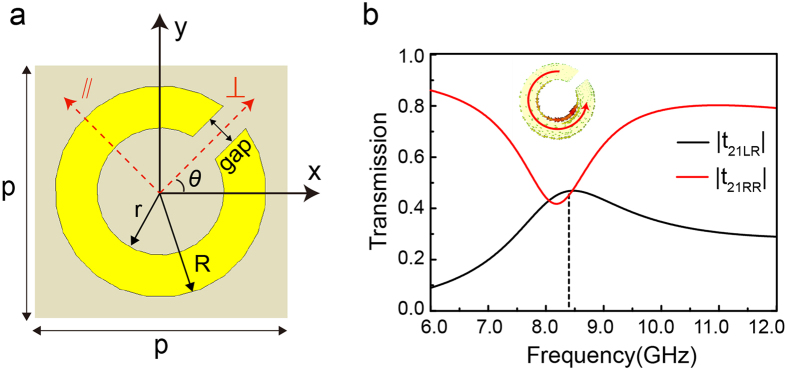
Single layer metasurface. (**a**) Schematic of metasurface with copper C-shape SRR (r = 1.5 mm, R = 2.5 mm, gap = 0.75 mm, p = 6.5 mm) patterned on FR4 substrate, where two optical axis are vertical ⊥ and parallel ∥ to the gap, respectively. *θ* is the rotation angle between ⊥ optical axis and x direction. (**b**) Simulated amplitude transmission of |*t*_21*LR*_| and |*t*_21*RR*_|, which are defined by the electric amplitude ratio between the output LCP or RCP waves and the input RCP wave, respectively. The inset displays the current distribution at the resonant frequency.

We design the metasurface with C-shaped SRRs patterned on FR4 substrate, as shown in Fig. 1a. To better understand the interaction between C-shaped SRR and CP wave, the electric field of CP wave is decomposed along two optical axises vertical and parallel to the gap, respectively, and the transmission coefficient is calculated based on Jones Matrix^34^,


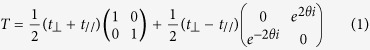


where *t*_⊥_, *t*_//_ are the transmission coefficients for the polarizations along two optical axis, and *θ* is the rotation angle between 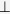 optical axis and *x* direction. According to Eq. (1), the co-polarization has amplitude of 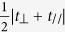 and phase same to the incident wave, while the cross-polarization has amplitude of 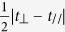 and phase shift of ±2*θ*, i.e. P-B phase, where the symbol ± is defined for the helicity of left-handed circularly polarized (LCP) and right-handed circularly polarized (RCP) incidence. Because of the contribution of P-B phase to reshape wavefront, the cross-polarized field is mainly focused on in this paper. Figure 1b shows the amplitude transmission |*t*_21*LR*_| of single-layer metasurface under normal RCP incidence. The amplitude transmission has a maximum of 0.5 similar to previous reports, which is attributed to an anti-symmetric electric resonance mode, as illustrated in surface current (inset in Fig. 1b). At such frequency, 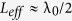, where 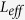 is the effective perimeter of SRR and λ_0_ is the resonant wave number. When the frequency deviates from the resonant frequency, the conversion efficiency further reduces.

To improve the low efficiency, multilayer structure is adopted in the designed metasurface array. It forms Fabry-Perot like cavity to produce constructive or destructive interference, which facilitates the well control of cross-polarization or co-polarization and promotes a high transmission as the phase difference ±2*θ* when CP wave pass through each layer metasurface. It is convenient to deal with multilayer metasurface by transfer matrix method (TMM) when the layer number is large. Hence, in addition to the finite element numerical simulation, we also conduct a theoretical calculation of TMM to characterize the transmission of CP wave through a multilayer metasurface, where a 4 × 4 transfer matrix is developed specially for CP wave (see Supplementary Information for details).

**Figure 2:**
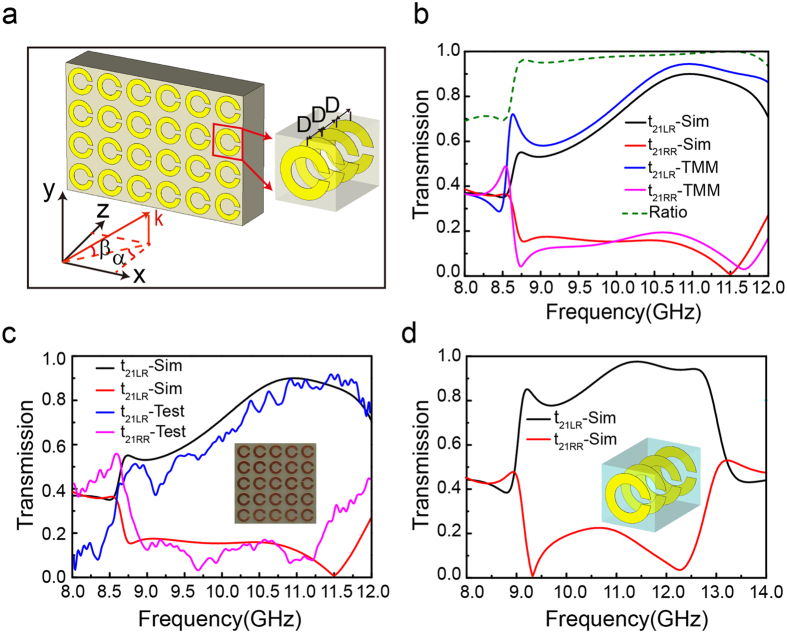
Four-layer metasurface arrays. (**a**) Schematic of four-layer metasurface arrays with an equal spacing D = 2.5 mm. (**b**) Simulated and calculated amplitude transmission, as well the polarization conversion ratio based on simulation. (**c**) Experimentally measured and simulated amplitude transmission, where the inset shows the photo of experimental sample. (**d**) Amplitude transmission for the metasurface array using Rogers substrate having geometry parameters r = 2.0 mm, R = 3.0 mm, gap = 0.75 mm, p = 7.0 mm, D = 3.0 mm.

Figure 2a shows the schematic diagram of a typical four-layer metasurface with Cu patterns on FR4 substrate, as the tradeoffs between bandwidth and high conversion efficiency (Supplementary Fig. S2). Figure 2b shows the simulated and calculated amplitude transmission of cross-polarization LCP wave and co-polarization RCP wave when a RCP wave is incident normally on the metasurface. It is noted that the simulated result agrees with the calculated one by TMM very well. The transmission coefficient of cross-polarization |*t*_21LR_| exceeds 80% within a broad band of 10.2–11.8 GHz and reaches the maximum of 90% at 11.0 GHz. On the contrary, the transmittance of co-polarization |*t*_21RR_| is much lower, so that the polarization conversion ratio 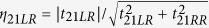 is as high as to be close to 1 over the almost whole X-band, indicating a pure CP wave output.

To confirm the above results of simulation and calculation, we fabricate the metasurface specimen accordingly and measure the transmission spectra under the normally incident RCP wave. As shown in Fig. 2c, the experiment results coincide with the simulated one very well. The amplitude transmission of |*t*_21*LR*_| is beyond 0.8 in a broad band of 10.3–11.7 GHz, while that of |*t*_21*RR*_| is below 0.2 within 8.5–11.2 GHz. It indicates that the proposed multilayer metasurface has high transmission efficiency within a broad bandwidth and a pure CP wave output. Further study shows that the metasurface array is not very sensitive to the incident angle and the polarization conversion efficiency maintains a high value within a wide incident angle range from 0° to 60° (Supplementary Fig. S3).

The transmission can be further improved by using dielectric substrate with lower permittivity and loss. If the FR4 substrate is replaced by Rogers 5880, the metasurface array exhibits much better performance (Fig. 2d). The amplitude transmission of |*t*_21*LR*_| exceeds 0.8 within an ultrabroad band of 9–13 GHz and the maximum even approach 1.

**Figure 3:**
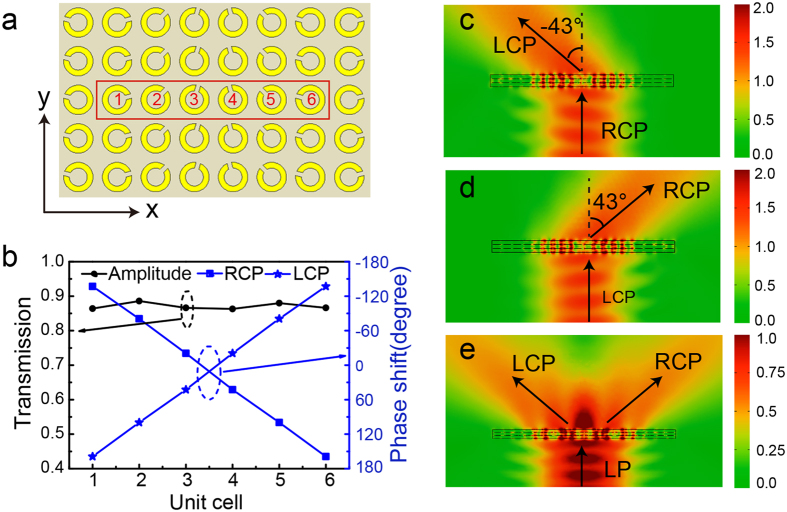
Simulations of polarized beam splitter at 11.0 GHz. (**a**) Front view of the polarized beam splitter consisting of super-unit cell with six scatters. (**b**) Amplitude and phase shift for six unit cells under the normally incident RCP and LCP waves. (**c**–**e**) Simulations of electric intensity distributions in the xz-plane under the normally incident RCP, LCP and LP Gaussian beams.

The high conversion efficiency, pure CP output and broad bandwidth can greatly enhance the performances of metasurface. Basically, the artificially designed metasurface arrays can efficiently control the P-B phase profile for polarization conversion. One of the most typical devices is the polarized beam splitter to bend transmitted wave to arbitrary direction by importing a gradient phase or a transverse momentum at the surface, where two conditions, the uniform amplitude for each scatter and a 0-2π phase coverage in an array period are required. We proposed a polarized beam splitter array consisting of super-unit cell with six scatters whose pattern rotates 30° than neighbor, as shown in Fig. 3a. Under the normally incident RCP and LCP waves, the amplitude (identical for RCP/LCP) of scattering field keeps uniform and their phase shifts linearly, as shown in Fig. 3b, which indicates that the polarized beam splitter has a good performance in abnormal transmission. The refractive angle can be deduced by the general Snell’s law^17^


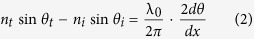


where *n*_*i*_ and *n*_*t*_ stand for the refractive indexes of input and output medium, 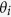 and 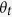 the angles of incidence and refraction, and *λ*_0_ the wavelength in vacuum. Figure 3c,d show the electric intensity distribution in the xz-plane under the normally incident LCP and RCP Gaussian beam with a 30 mm beam waist, respectively. It is clear that the transmitted wave has similar intensity as the incident wave, indicating a high transmission efficiency which is about 80% by radiation pattern. A little reflection is mainly due to the change on wave impedance compared with the homogenous metasurface array^35^. Different handedness of incident CP wave produces opposite phase shift and changes the trajectory of transmission, which offers another freedom to manipulate CP wave. According to Eq. (2), the theoretical refractive angle is calculated as 
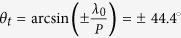
 for LCP and RCP incidences, which agrees with the simulated results of ±43°. The well-made gradient metasurface specimen exhibits transmission performance matching with the calculated and simulated results very well. Besides, if a LP wave is incident on the gradient metasurface array, the transmitted wave is separated into two directions symmetrically, as shown in the Fig. 3e. It is because LP wave can be decomposed into two CP components with opposite handedness and uniform amplitude.

**Figure 4:**
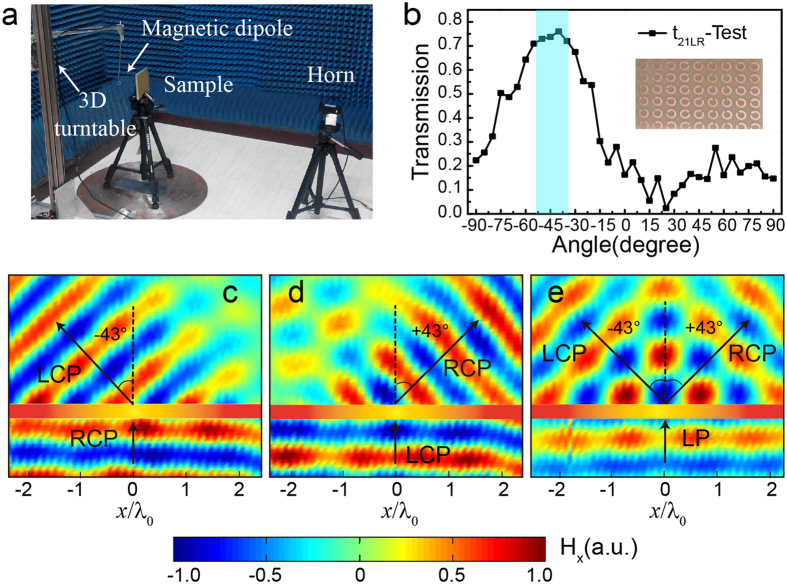
Measurements of fabricated polarized beam splitter at 11.0 GHz. (**a**) Setup of the 3D near field scanning system in an anechoic chamber. (**b**) Experimentally measured amplitude transmission |*t*_21*LR*_| vs. detecting angles. The inset shows the photo of experimental sample. (**c**–**e**) Measured distribution of transmitted magnetic field H_x_ in the xz-plane under the normally incident RCP, LCP, LP plane wave.

The experimental measurements of polarized beam splitter are also conducted by 3D near field scanning system in anechoic chamber (Fig. 4a). As shown in Fig. 4b, the maximum amplitude of transmitted wave appears at about −45° with an amplitude transmission |*t*_21*LR*_| ~80% when RCP approximate-plane-wave is incident normally on the metasurface array. Figure 4c–e are the measured distribution of magnetic field H_x_ in the xz-plane under the normal LCP, RCP, LP incidence at 11.0 GHz. The angles of refraction are ±43°, respectively, which are well corresponding to the results of simulation and theory calculation. Little deviation can be eliminated by adding a pair of lens between feed horn and sample^36^, to obtain a better approximating plane wave excitation and avoiding edge diffraction scattering.

**Figure 5:**
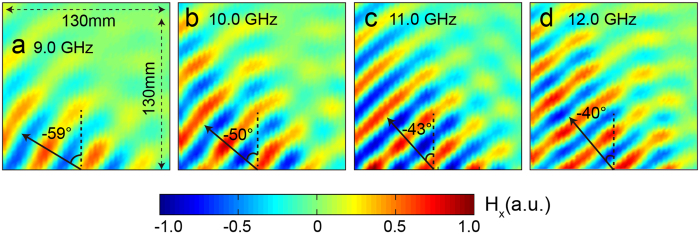
Measurements of fabricated polarized beam splitter at different frequencies. (**a**–**d**) Measured distribution of transmitted magnetic field H_x_ in the xz-plane under the normally incident RCP plane wave at 9.0 GHz, 10.0 GHz, 11.0 GHz, 12.0 GHz.

Because the P-B phase is independent of incident wavelength, the gradient metasurface array has a good performance within a broad bandwidth. Figure 5a–d show the measurement distributions of transmitted magnetic field H_x_ in the xz-plane under the normal RCP incidence at 9.0 GHz, 11.0 GHz, 11.0 GHz and 12.0 GHz. The measured refractive angles are −59°, −50°, −43°and −40°, respectively, agreeing well with the theoretical values −58.7°, −50.3°, −44.3°, −39.9°calculated by Eq. (2). Besides, the transmission conversion efficiency can be further improved in a more broad bandwidth (Supplementary Fig. S4) if the FR4 substrate is replaced by low loss Rogers and the maximum of conversion efficiency reaches 92%.

## Discussion

We proposed and fabricated multilayer metasurface consisting of classic C-shaped SRR. The theoretical calculation, numerical simulation and microwave experiment at X-band all confirm that the metasurface array has high cross-polarization conversion efficiency over a broad bandwidth. Full control of P-B phase in such metasurface brings diverse functions, which well inherit the high efficiency and broad bandwidth. A gradient metasurface array is designed and fabricated to demonstrate the abnormal transmission. With full capacities of high transmission efficiency of polarization conversion, broad bandwidth and high purity of CP wave output, the proposed multilayer metasurface arrays have promising future in many applications, such as optical interconnection, optical alignment, beam splitter and on-chip optical or logic component. Moreover, the proposed metasurface can be also extended to the manipulation of LP wave with high efficiency, which provide wider application areas.

## Methods

### Simulation

All the numerical simulations are calculated by Radio Frequency (RF) Module of COMSOL Multiphysics ver. 4.4. For the period structure, a single unit cell is simulated with periodic boundary conditions and wave ports in x, y and z directions, respectively. However, the beam splitter is modeled in the vacuum environment where perfectly matched layers and periodic boundary conditions are in x, z and y directions, respectively. The excitations are Gaussian beams with a 30 mm beam waist and propagating along z direction. The dielectric constant of FR4 (Rogers 5880) is 4.0 (2.1) with a loss tangent 0.015 (0.001). The copper film is simulated with a thickness of 0.035 mm and a default conductivity of 5.99 × 10^7^ S/m.

### Fabrication and measurement

All the metasurface specimens are fabricated by standard printed circuit board (PCB) technology and characterized in the anechoic chamber. The transmission parameters are measured by a couple of CP horns (8–12 GHz) connected to the transmitted and received ports of a vector network analyzer (Agilent 8722ES). The sample is in the middle of two horns whose distance is about 1 m to obtain an approximating plane wave. The measurements of abnormal refraction and magnetic field distributions are conducted by 3D near field scanning system, as shown in Fig. 4a. An electrically small shielded loop (magnetic dipole) with a diameter of 5 mm or a CP horn connecting to receive port of Agilent 8722ES is mounted a 3D turntable whose position is controlled by a stepper motor and record the data with a 2 mm × 2 mm resolution in the xz-plane.

## Additional Information

**How to cite this article**: Liu, C. *et al*. Fully Controllable Pancharatnam-Berry Metasurface Array with High Conversion Efficiency and Broad Bandwidth. *Sci. Rep.* **6**, 34819; doi: 10.1038/srep34819 (2016).

## Supplementary Material

Supplementary Information
