# Supplementary material for: Fully Controllable Pancharatnam-Berry Metasurface Array with High Conversion Efficiency and Broad Bandwidth
Source: Sci Rep. 2016 Oct 5;6:34819. doi: 10.1038/srep34819 (PMC5050500; doi:10.1038/srep34819)
Supplement: Supplementary Information [file srep34819-s1.doc]

# Supplementary Information

# Fully Controllable Pancharatnam-Berry Metasurface Array with High Conversion Efficiency and Broad Bandwidth

Chuanbao Liu^1^, Yang Bai^1,*^, Qian Zhao^2^, Yihao Yang^3^, Hongsheng Chen^3^,

Ji Zhou^4^, and Lijie Qiao^1^

^1^Key Laboratory of Environmental Fracture (Ministry of Education), University of Science and Technology Beijing, Beijing 100083, China

^2^ State Kay Laboratory of Tribology, Department of Mechanical Engineering, Tsinghua University, Beijing 100084, China

^3^State Key Laboratory for Modern Optical Instrumentation, Zhejiang University, Hangzhou 310027, China

^4^State Key Laboratory of New Ceramics and Fine Processing, Tsinghua University, Beijing 100084, China

^*^Author to whom correspondence should be addressed.

Electronic mail: [baiy@mater.ustb.edu.cn](mailto:baiy@mater.ustb.edu.cn)

## Transfer matrix method for CP wave

As partial scattered fields happen to polarization rotation, a 4×4 transfer matrix is developed specially for CP wave. The metasurface is assumed between two dielectric layers 1 and 2. A 4×4 transfer matrix *M*_21_ related to the forward and backward fields in the both sides of dielectric layers (Fig. S1) is utilized due to the polarization conversion and can be expressed as:

$$\left(\begin{matrix} E_{2R}^{f} \\ E_{2L}^{f} \\ E_{2L}^{b} \\ E_{2R}^{b} \end{matrix}\right)=M_{21}\left(\begin{matrix} E_{1R}^{f} \\ E_{1L}^{f} \\ E_{1L}^{b} \\ E_{1R}^{b} \end{matrix}\right)$$

where the superscript *f* and *b* mean forward and backward EMWs. The transfer matrix *M*_21_ can be expressed in terms of reflection coefficient *r* and transmission coefficient *t*:$M_{21}=\left(\begin{matrix} 1 & 0 & -r_{22RL} & -r_{22RR} \\ 0 & 1 & -r_{22LL} & -r_{22LR} \\ 0 & 0 & t_{12LL} & t_{12LR} \\ 0 & 0 & t_{12RL} & t_{12RR} \end{matrix}\right)^{-1}\left(\begin{matrix} t_{21RR} & t_{21RL} & 0 & 0 \\ t_{21LR} & t_{21LL} & 0 & 0 \\ -r_{11LR} & -r_{11LL} & 1 & 0 \\ -r_{11RR} & -r_{11RL} & 0 & 1 \end{matrix}\right)$

For the multilayer structure, we first acquire the transfer matrix of each metasurface and then solve the total transfer matrix *M_total_* by the following equation:

where *P* is a diagonal matrix$P=diag(\mathrm{e}^{ik_{0}nd},\mathrm{e}^{ik_{0}nd},\mathrm{e}^{-ik_{0}nd},\mathrm{e}^{-ik_{0}nd})$, *k*_0_ is the vacuum wave number, *n* is the refraction index of dielectric and *d* is the thickness of dielectric.


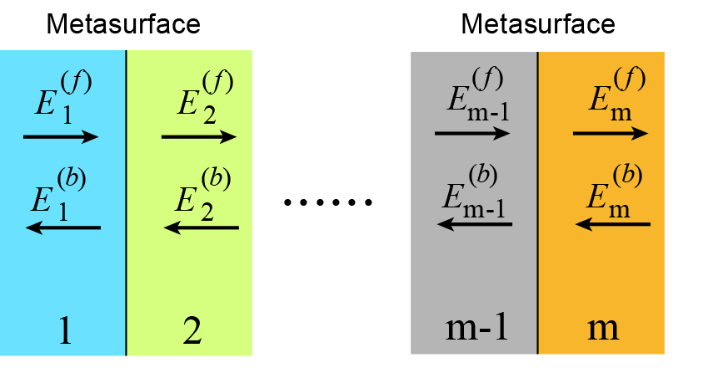


Fig. S1. Schematic of transfer matrix model with forward and backward EW fields in mediums.

## The selection of multi-layer structure

For multi-layer structure, the geometry parameters and materials are same as those in Fig. 1a, and the spacing D between adjacent layers equals 2.5 mm. The simulated results (Fig. S2) show that four-layer metasurface arrays are the best choose to meet the requirements of high polarization conversion efficiency and broad bandwidth.

**
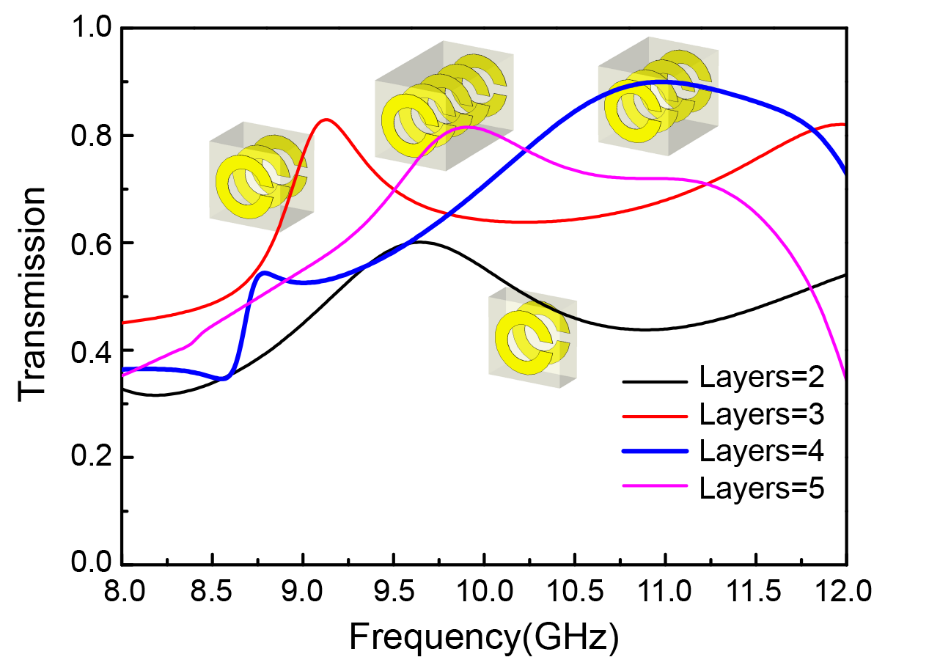
**

Fig. S2. Amplitude transmission $\left|t_{21LR}\right|$for metasurface arrays with different layers under normally incident RCP wave.

## Influence of incident angles on the amplitude transmission

For the four-layer metasurface arrays, we study the incident angle α (β=0) and β (α=0) from 0° to 75°, as shown in Fig. S3a and Fig. S3b, respectively. The results demonstrate insensitive feature to incident angles.


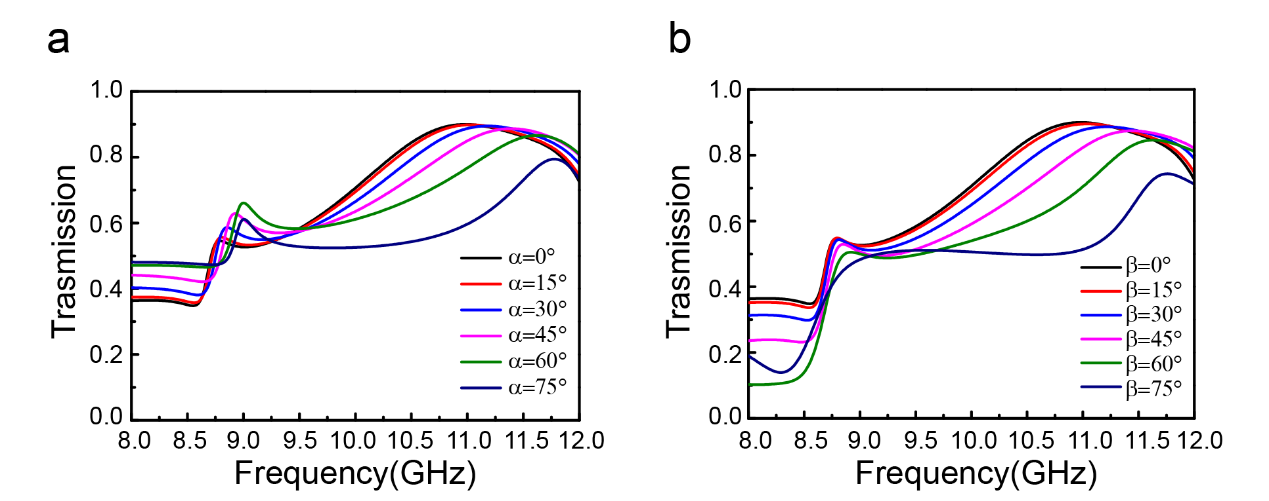


Fig. S3. Amplitude transmission $\left|t_{21LR}\right|$for four-layer metasurface array under RCP wave incident from different angles.

## Electric intensity distribution for different frequencies

Rogers structure was adopted for better demonstrating the characteristics of broad operation bandwidth and high conversion efficiency. Constituting scatters have same geometry parameters as those in the inset of Fig. 2d. The results show that the abnormal refraction is valid within a broad frequency range, where only the transmission efficiency changes slightly, as shown in Fig. S4a-d.


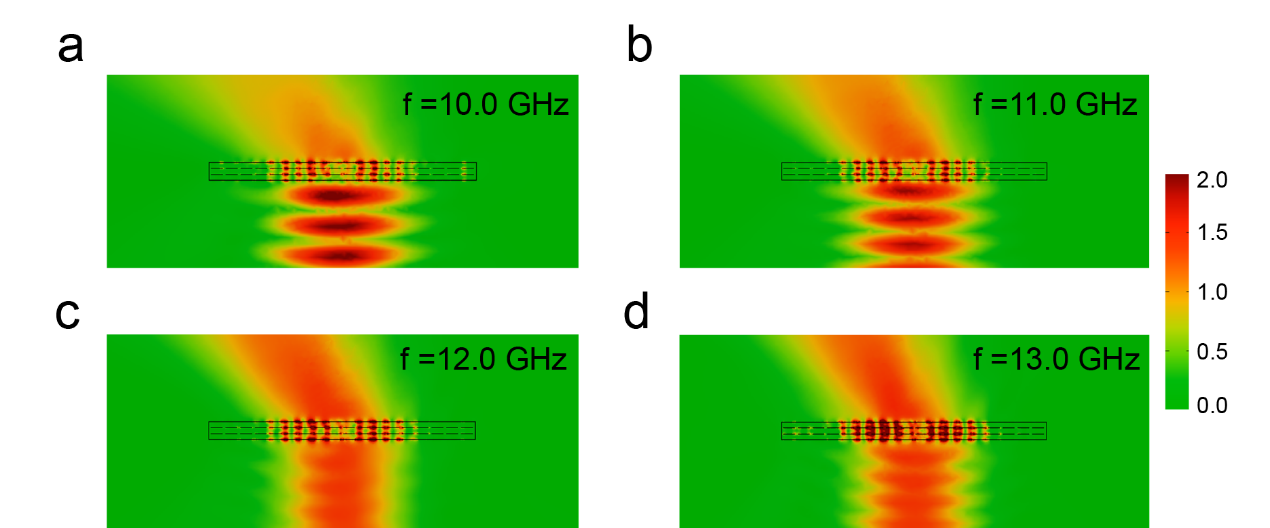


Fig. S4. **(**a)-(d) Electric intensity distribution in the xz-plane from 10.0 to 13.0 GHz under the normally incident RCP Gaussian beam.
